# Hidden *Staphylococcus aureus* Carriage: Overrated or Underappreciated?

**DOI:** 10.1128/mBio.00079-16

**Published:** 2016-02-16

**Authors:** Alex van Belkum

**Affiliations:** bioMérieux, Microbiology R&D, La Balme Les Grottes, France

## Abstract

*Staphylococcus aureus* is a persistent companion bacterial species in one-third of humankind. Reservoirs include the nasal and nasopharyngeal cavities, skin, and gastrointestinal (GI) tract. Despite earlier claims that colonization of individuals is caused by clonal organisms, next-generation sequencing (NGS) has revealed that resident type heterogeneity is not exceptional. Carriage, whether overt or hidden, is correlated with a risk of autoinfection. In a recent article in *mBio*, it was shown that, based on staphylococcal genome sequencing, low-level GI persistence may cause long-term nosocomial outbreaks [L. Senn et al., 7(1):e02039-15, 2016, doi:10.1128/mBio.02039-15]. Institutional endemicity with methicillin-resistant *S*. *aureus* (MRSA) sequence type 228 (ST228) is shown to originate not from high-level nasal carriage or poor compliance with infection control practice but from low-grade asymptomatic GI colonization. This shows the power of NGS in elucidating staphylococcal epidemiology and, even more important, demonstrates that (drug-resistant) microorganisms may possess stealthy means of persistence. Identifying these persistence mechanisms is key to successful infection control.

## HUMAN CARRIAGE OF *S. AUREUS* AND CONSEQUENCES FOR INFECTION CONTROL

*Staphylococcus aureus* colonizes humans and has a preference for the nasopharyngeal area, skin, and gastrointestinal (GI) tract, including the perineum. Local microbiota are important determinants of strain colonization of a specific host, and competitive interactions with *Corynebacterium* species and coagulase-negative staphylococci have been observed on various occasions. Relatively little is known about the host and microbial factors driving niche specificity, although many suggest the importance of microbial surface components recognizing adhesive matrix molecules (MSCRAMMs) on host surfaces (1). Obviously, additional host factors, including cellular and humoral immunity, play important roles. However, determining niche colonization preferences of staphylococcal strains (the nasal niche versus the gastrointestinal niche) remains elusive.

Most of the studies relating staphylococcal colonization and infection risk have focused on nasal carriage, since it is still assumed that the nose is the primary staphylococcal niche. A limited number of studies have shown a clear correlation between nasal carriage and, for instance, post-surgical wound infection, where some studies have even shown that presurgical elimination of *S. aureus* carriage protects against such infection. However, most of these studies have been quite biased toward nasal carriage, and limited data exist on the importance of GI colonization (2). Additional studies on colonization dynamics are still needed.

## NEW TECHNOLOGY FOR DEFINITION OF *S. AUREUS* CARRIAGE AND TRANSMISSION PATHWAYS

All studies focusing on infection control or nosocomial epidemiology of *S. aureus* strains depend on the resolving power of the technology used for bacterial typing. The more stringent the technology, the stronger the correlative claims between carriage and autoinfection. Using simple typing technologies, most of today’s *S. aureus* epidemiology knowledge was defined in the 1960s. Some argue that what we do today is repeat previous studies, simply using newer technologies. However, the new generation of “omics” technologies provides the data that allow for unprecedented detail in epidemiological studies as such.

The prime concern regarding previously published studies is the resolution of the methods used. Undoubtedly, screening a few dozen restriction sites using pulsed-field gel electrophoresis (PFGE) may show a different degree of relatedness among strains than the nucleotide sequence-based analysis of repetitive DNA in the protein A gene (*spa*). A continuous technological evolution in the typing field has resulted in advocation for the next technology as at least complementary, but often clearly better than, its predecessors. The recent emergence of a variety of “omics” technologies has generated huge databases that enable detailed comparison of sets of microbial isolates. Although discussion on the most-optimal “omic” technology persists, there is a strong argument that bacterial whole-genome sequencing (WGS) using next-generation sequencing (NGS) technology might provide a universal solution (3). Comparisons of the 2.5- to 3.0-Mbp *S. aureus* genomes can be focused on the core genome, variable genome, and even mobile genetic elements, which gives a way to restrict diversity to a level representative of phylogeny or recent epidemiological relatedness with contemporary and ancestral isolates encountered in the same environment (e.g., the hospital or nursing home). On the basis of full-genome comparisons, it has been shown that evolution of *S. aureus* proceeds with between 1 and 4 nucleotides per 10^6^ bp per year (4). Similar values for strains that transfer from colonization to infection or from host to host are still quite ill-defined. Dedicated software packages will help interpret the huge data sets and derive species identification, strain types, antibiograms, and virulence properties directly from bacterial sequence motifs obtained from clinical specimens (5). Technically, the stage has now been set for definitive bacterial strain typing.

## RELEVANCE OF COVERT *S. AUREUS* CARRIAGE

In a recent article in *mBio*, Senn et al. used NGS to address a long-term outbreak with *S. aureus* sequence type 228 (ST228) in a Swiss hospital (6). They sequenced a reasonable fraction of the isolates that were obtained from 1,600 patients over a period of 4 years. They demonstrated persistence of a single clone, and by combining genome data with epidemiological and patient data, they showed that this particular clone prefers the rectum and groin over the nasal cavity as primary colonization niches. In addition, their data show that unrecognized carriers of the methicillin-resistant *Staphylococcus aureus* (MRSA) clone have played a major role in the persistence and spread of this particular strain. What Senn et al. define as “stealthy colonization of the gut” has probably facilitated this outbreak, conceivably in combination with heightened transmissibility.

These data shed new light upon the relevance of gut colonization by *S. aureus* and its implications for hospital epidemiology of MRSA. In several countries, patients will be isolated upon nasal carriage with MRSA; the question arises whether the same strictness should be imposed for GI carriage. The question is also whether tests that are currently used for detecting nasal carriage can detect gut carriage. Culture and molecular methods each have their own pitfalls, and most tests have primarily been evaluated using nasal swabs. The Swiss work (6) shows that GI carriage likely involved relatively small numbers of staphylococcal cells, which may affect detection capabilities. Whether this lower density reflects a decreased staphylococcal colonization capacity or a colonization resistance effect of the local microbiota (and possibly host factors) is another question that still remains unanswered. It is unknown whether the gene content of GI colonizers is significantly different from those of “optimal nasal colonizers.” The data analysis by Senn et al. (6) is quite selectively focused on the core genome, whereas these questions will require a full-genome approach.

## FUTURE PERSPECTIVE

Large-scale and long-term studies as presented by Senn and colleagues (6) are important, since these types of studies allow for the precise assessment of (new) epidemiological and biological scenarios. As shown here, NGS is suited for the elucidation of new mechanisms of bacterial spread and clearly shows the differential preferences of bacterial strains for specific anatomical locations. However, in order for NGS methods to become really useful from a hospital infection control perspective, methods need to be accelerated and rendered much cheaper still. Bacterial typing is useful only when acting as close to prospective as possible, since only then can timely preventive measures be developed. Sequence quality should be guaranteed and maybe even FDA validated, the sequencing technology should fit within a working day, and interactive software should produce clear and simple outputs to guide the infection control team. In order to provide these proposed measures, we must fill major gaps needed for basic genomic calibration as follows.

• How many mutations should we allow to occur between strains before calling them epidemiologically unrelated?

• What is the basic mutation frequency during colonization of a host?

• What happens if the strain moves from one host species to another?

• Which mutation and how many mutations are fixed by bottlenecking when one strain transfers to a new host or transits from colonization to infection?

• Do single-nucleotide mutations have impacts similar to those of complete gene deletions and are silent mutations ever relevant (e.g., at the regulation level)?

• Are mobile genetic elements relevant, since it has already been shown that epidemics can be driven by such elements, rather than a bacterial clone?

• Are there gene families that are more often affected than others and do mutations in such families provide selective advantages for the strains involved?

• What is the overall effect of antibiotics and biocides on mutational frequency or strain fitness?

• How do strains behave when present in different types of microbiota and have to compete or cooperate with different mixtures of other bacterial species?

• How do strains evolve outside their optimal ecological niche (e.g., in the environment)?

The above need to be quantified on different time scales, ranging from days to even years, with these data taken into account when setting up interpretative guidelines for the correct interpretation of sequence diversity.

Regarding the issues listed above, there are still abundant biological and genetic questions to be answered, with studies like the one presented by Senn et al. (6) paving the way. The answers will vary widely when different species are concerned, but this will ultimately enrich our knowledge on genome polymorphisms, ecological behavior, and host specificity of human microbial pathogens.

A final issue remains that cannot be avoided when considering application of NGS technologies. Affordability remains an important issue with current NGS. In a setting where even “freely” available antibiograms do not have an impact on infection control activities, sequencing will simply be considered too costly or too complex.

In the assessment of the epidemiology of hospital-acquired infections (HAI), the NGS studies have been solely retrospective thus far. By addressing issues discussed here, NGS studies may move to being genuine drivers of prospective intervention.
